# Nitric Oxide Regulates Multiple Signal Pathways in Plants via Protein *S*-Nitrosylation

**DOI:** 10.3390/cimb47060407

**Published:** 2025-05-30

**Authors:** Wei Lin, Jian-Xiu Shang, Xiao-Ying Li, Xue-Feng Zhou, Li-Qun Zhao

**Affiliations:** 1 School of Biology and Food Science, Hebei Normal University for Nationalities, Chengde 067000, China; 15630185650@163.com (W.L.); 19133613371@163.com (X.-F.Z.); 2 Key Laboratory of Molecular and Cellular Biology of Ministry of Education, College of Life Sciences, Hebei Normal University, Shijiazhuang 050024, China; shangjianxiu@mail.hebtu.edu.cn (J.-X.S.); xying0506@163.com (X.-Y.L.)

**Keywords:** growth and development, nitric oxide, *S*-nitrosylation, stress response

## Abstract

Nitric oxide (NO) can perform its physiological role through protein *S*-nitrosylation, a redox-based post-translational modification (PTM). This review details the specific molecular mechanisms and current detection technologies of *S*-nitrosylation. It also comprehensively synthesizes emerging evidence of *S*-nitrosylation roles in plant biological processes, including growth and development, immune signaling, stress responses and symbiotic nitrogen fixation. Furthermore, the review analyzes research progress on the crosstalk between *S*-nitrosylation and other protein PTMs. Finally, unresolved issues such as the spatio-temporal resolution of SNO-proteome mapping and standardized protocols for reproducibility are pointed out. In summary, this work proposes a roadmap for future research.

## 1. Nitric Oxide and *S*-Nitrosylation

Key roles for nitric oxide (NO) in plants have been demonstrated in seed dormancy, embryogenic cell formation, root development and gravitropic bending, flowering, stomatal closure, the growth regulation of pollen tubes, nutrition (particularly ion homeostasis), immunity, and adaptive responses to various abiotic stresses [1,2]. For instance, NO operates through reactive oxygen species (ROS) and classical second messengers, including Ca^2+^ and cyclic guanosine monophosphate, to impact physiological processes in plants [3,4]. Further, crosstalk exists between NO and key hormones, including auxin, abscisic acid (ABA), salicylic acid (SA), jasmonic acid (JA), ethylene, and cytokinins in plant metabolism [5]. However, the molecular mechanisms whereby NO impacts the plant life cycle are poorly understood.

In recent years, *S*-nitrosylation has emerged as an important NO-dependent protein post-translational modification (PTM) involved in a large variety of cellular functions [6]. During *S*-nitrosylation, an NO group is covalently linked to the free sulfhydryl group of specific cysteine (Cys) residues in the targeted protein, forming *S*-nitrosothiol (SNO) [7]. SNO is dynamically labile in response to the intracellular redox status, making *S*-nitrosylation a highly sensitive mediator of cell signaling [8]. The factors that influence the specific formation of SNO modifications include the redox state of cells or tissues, the subcellular localization and acid–base environment of Cys residues, the local NO concentration and the expression levels of transnitrosylase or denitrosylase [9]. The efficiency of *S*-nitrosylation is also influenced by the concentration of intracellular ROS and NO radicals [10]. Cys residues with acid–base motifs, high sulfur-atom exposure space and low pKa are more susceptible to SNO modification [11]. It has been reported that the sequence of EXC (where E represents Glu, X indicates any amino acid residue, and C represents Cys) is a putative consensus sequence of *S*-nitrosylation protein, which has a higher probability of the modification [12].

**Figure 1:**
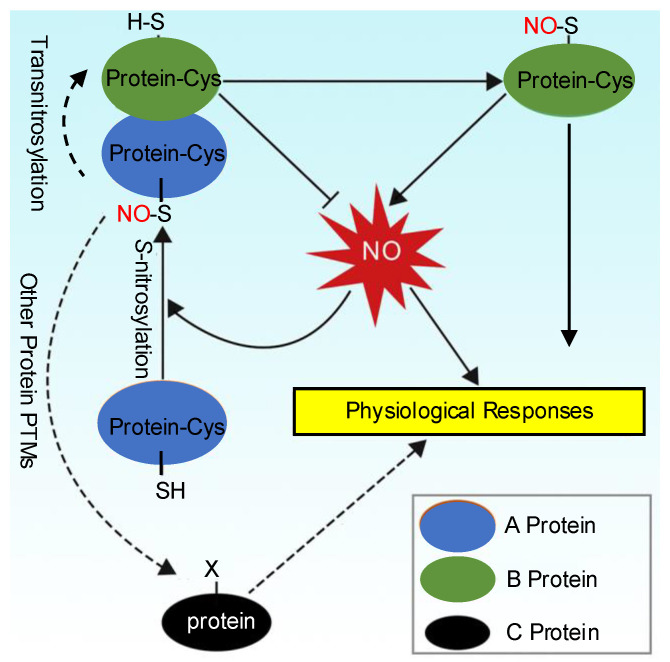
A proposed model of NO-mediated *S*-nitrosylation signaling. NO induces *S*-nitrosylation of A protein and the *S*-nitrosylated A (A-SNO) may subsequently transnitrosylate B at cysteine residues. A-SNO may also participate in regulation of other protein PTM. The NO-mediated *S*-nitrosylation regulates various physiological through different pathways.

Due to the development of dedicated proteomic approaches, especially the fluorous affinity tag (FAT)-switch method combined with mass spectrometry (MS), hundreds of *S*-nitrosylated proteins have been identified in plants [13]. Moreover, *S*-nitrosylation is accepted as a cell signaling mechanism with important functional implications in various physiological processes [6,14]. The diagram that illustrates NO-mediated *S*-nitrosylation signaling pathways in plant physiological processes is shown in Figure 1.

## 2. Identification of *S*-Nitrosylated Proteins in Plants: Methodological Aspects

### 2.1. The Biotin-Switch Technique

**Figure 2:**
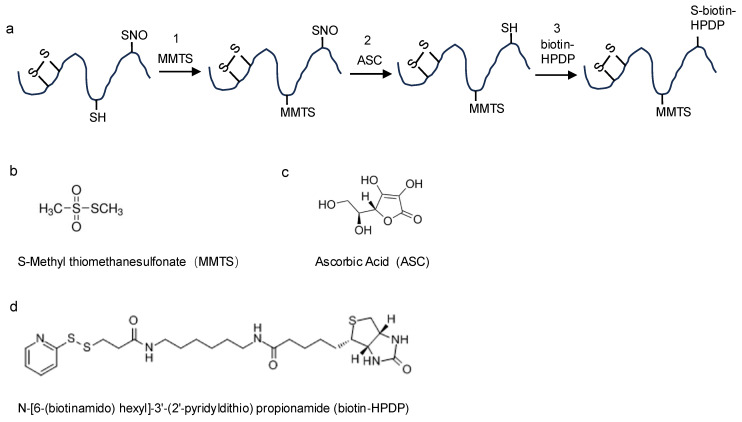
The schematic diagram of biotin-switch technique (BST) for detection of *S*-nitrosylated proteins. (**a**) Reaction steps for labeling *S*-nitrosylated proteins. 1, Unmodified Cys residues are blocked by MMTS. 2, The ASC specifically reduces *S*-nitrosylated Cys residues. 3, The newly liberated thiols are substituted by biotin-HPDP. (**b**) The structural formula of MMTS. (**c**) The structural formula of ASC. (**d**) The structural formula of biotin-HPDP.

The biotin-switch technique (BST) is an efficient tool for identifying *S*-nitrosylated proteins [15,16]. The method consists of three main steps (Figure 2). First, *S*-nitrosylated proteins from extracts or recombinants are treated with *S*-methyl methanethiosulfonate (MMTS) and 2.5% SDS at 50 °C for 30 min with recurrent vortexing to block unmodified Cys residues. Then, MMTS is removed by protein sedimentation with cold acetone, and the proteins are dissolved in RB buffer (25 mM HEPES, 1 mM EDTA, and 1% SDS, pH 7.7). Second, ascorbic acid (ASC) is added to the RB buffer and the mixture is incubated at room temperature for 1 h with recurrent vortexing in the dark. During this step, *S*-nitrosylated Cys residues are transformed into free Cys thiols by the reducing role of ASC. Because ASC is a poor reducing agent, long incubation times (up to 3 h) and high ASC concentrations (30 mM or more) can greatly enhance detection sensitivity [17]. Third, the free thiols (i.e., originally *S*-nitrosylated sites) are labeled with a sulfhydryl-specific biotinylating reagent, such as N-[6-(biotinamido) hexyl]-3′-(2′-pyridyldithio) propionamide (biotin-HPDP). Notably, steps 2 and 3 occur simultaneously to ensure the immediate biotinylation of the freshly generated thiols. Since MMTS groups decompose when exposed to light, all steps must be performed in complete darkness.

After the reactions are finished, the method used to identify *S*-nitrosylated proteins in plant extracts is different from that for recombinant proteins. In vivo, proteins labelled with biotinylated are purified by immunoprecipitation with streptavidin beads 12 h at 4 °C. Then, the beads are washed three times with HEN buffer (1.25 mM HEPES, 500 mM EDTA, and 1 mM neocuproine, pH 7.7). The bound proteins are eluted with 10 mM DTT in SDS-PAGE solubilization buffer then subjected to 10% SDS-PAGE at first. Then, the proteins are transferred to a polyvinylidene difluoride membrane for identification using appropriate antibodies. On the contrary, in vitro biotinylated proteins in SDS-PAGE solubilization buffer are firstly subjected to 12% SDS-PAGE. Then, the labeled proteins are analyzed by immunoblotting with the corresponding antibodies or liquid chromatography-tandem MS (LC-MS/MS). Detailed methods of LC-MS/MS detection are described in Section 2.3.

### 2.2. Other Methods Used to Detect S-Nitrosopeptides

It is difficult for biotin-related reagents to elute from the capture resin, which complicates MS/MS spectral interpretation. Therefore, only several hundred *S*-nitrosylated proteins have been detected by the BST in plants, limiting the further study of *S*-nitrosylation. Thus, it is important to find new reagents to label sulfhydryl groups. In recent years, several other labeling reagents, including isotope-coded affinity tags [18], isobaric iodo-TMT tags [19,20], Cys-specific phosphonate adaptable tags [21], bioorthogonal cleavable-linker tags [22], thiol-reactive resin tags [23], and FATs [12] have been found to label and enrich *S*-nitrosopeptides. These labels are used instead of thiol-reactive biotin in the third step of the process, while the rest of the procedure is similar to the BST. Given the lack of commercial antibodies that can recognize these labels, tag-switch-enriched proteins cannot be subjected to immunoblotting, which limits their application in proteomic studies.

Among these regents, FATs have covalent C–F bonds that prevent unexpected dissociation during MS/MS and reduce the complexity of MS/MS, thus allowing for the identification of low-abundance targets [12]. FATs have been successfully applied to proteomic studies of PTMs, including protein phosphorylation, tyrosine nitration, 4-hydroxy-2-nonenal modification, and *S*-nitrosylation [24,25]. Recently, Qin et al. (2023) developed an N-(4,4,5,5,6,6,7,7,8,8,9,9,9-trideca-fluorononyl) iodoacetamide tag-based FAT-switch approach, which showed a significantly improved sensitivity and efficiency of enrichment and a greater detection of *S*-nitrosylated peptides compared to the classic BST [13]. Thus, the FAT-switch method can be used for broadscale *S*-nitrosylation proteomic analyses. The comparations of BST and FAT-switch techniques are shown in Table 1.

Furthermore, the protein microarray-based approach can investigate low-mass *S*-nitrosothiols (SNOs) [26]. The numbers of *S*-nitrosylated Cys residues can be measured by DAN (NO fluorescence probe; Dimethyl diacetoxyfumarate) assay. In this method, the released NO from the thiol group is detected by fluorescence labeling [27].

### 2.3. LC-MS/MS

**Figure 3:**
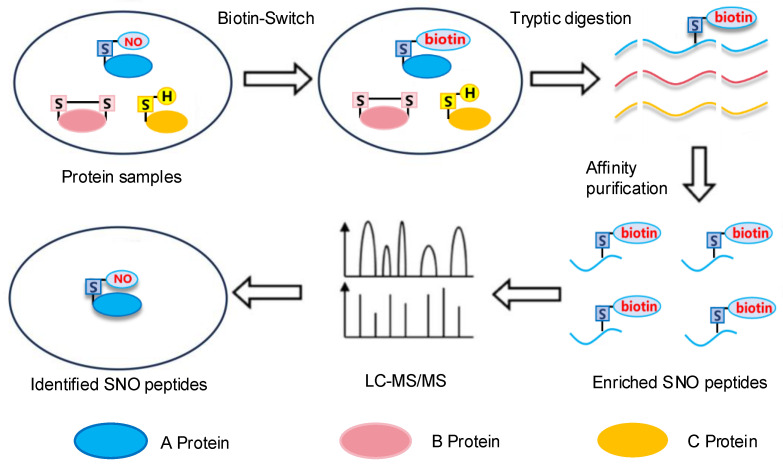
Schematic illustration of the procedure of site-specific *S*-nitrosylation analysis by LC-MS/MS.

Biotinylated proteins are analyzed by LC-MS/MS to identify *S*-nitrosylated residues [28]. LC-MS/MS involves the analysis of ionized molecules to determine their structure, molecular weight, and abundance. Schematic illustration of the procedure of site-specific *S*-nitrosylation analysis by LC-MS/MS is shown in Figure 3. In biology, two ionization techniques are mainly used: matrix-assisted laser desorption ionization (MALDI) and electrospray ionization (ESI). Both MALDI and ESI allow proteins or peptides to be ionized with high sensitivity [29,30]. Importantly, MALDI is less affected by salt impurities, and it is commonly chosen for analyzing peptides obtained from electrophoresis gels through peptide mass fingerprinting.

Based on the mass-to-charge change of a specific PTM, LC-MS/MS can be effectively used to detect and identify the modification. Importantly, MS/MS experiments are required to confirm that the mass shift detected in the original ion is also found in the fragment ions carrying the labelled amino acid residue. During the application of BST, *S*-nitrosylated Cys residues can be detected by ionization after biotinylation. The labelled peptides are separated, enriched, and identified by MALDI to find an addition of 428 Da [31].

To identify proteins, MS/MS spectra made by LC-ESI-MS are updated in the BioTools 2.0 platform to search the NCBInr database using a licensed version of the Mascot v.2.2.04 search engine (www.matrixscience.com, accessed on 20 March 2025; Matrix Science, Israel, India, South Korea, et al.). For reduced samples, carbamidomethyl Cys is defined as a fixed modification from treatment with iodoacetamide, but oxidized methionine is regarded as a variable modification. Additional settings include a peptide mass tolerance of 0.5 Da for the parental ion mass and fragment ion masses and one missed cleavage site. For non-reduced samples, biotin-HPDP Cys modification is defined as a variable modification.

## 3. Functions of *S*-Nitrosylation in Plants

NO performs its physiological role primarily through protein *S*-nitrosylation, a redox-based PTM that is largely determined by the local concentration of NO and the structure of the modified protein [32,33,34]. *S*-nitrosylation is involved in various signaling pathways, including those affecting plant growth, development, immune responses, stress responses and symbiotic nitrogen fixation [6].

### 3.1. Growth and Development

NO-mediated *S*-nitrosylation participates in multiple phases of plant growth and development through regulating hormone signaling [35,36,37]. The functions of *S*-nitrosylated proteins related to growth and development are summarized in Table 2.

#### 3.1.1. ABA Signaling

ABA is involved in seed germination and early seedling growth [38]. The application of exogenous NO breaks seed dormancy and alleviates the inhibitory effects of ABA on seed germination and early seedling growth [39,40]. For example, NO induces the *S*-nitrosylation of SnRK2.6 at Cys-137 and decreases the kinase activity of SnRK2.6, thereby inhibiting ABA signaling and promoting seed germination [41]. ABA insensitive5 (ABI5) is the core transcription factor that represses seed germination in ABA signaling. The *S*-nitrosylation of ABI5 promotes seed germination and seedling growth through triggering ABI5 degradation [42,43]. MYB30 triggers an NO-induced decrease in ABA content during germination [44]. The *S*-nitrosylation of MYB30 controls seed dormancy and germination by enhancing its transcriptional activity in *Arabidopsis thaliana* (Arabidopsis) [45]. These findings show that *S*-nitrosylation is important for seed dormancy and germination.

#### 3.1.2. Auxin Signaling

NO-mediated *S*-nitrosylation regulates plant growth and development through auxin signaling [16,46,47]. For example, the *S*-nitrosylation of ROP2 reduces the rate of auxin transport by changing the intracellular localization of ROP2, thereby inhibiting root growth in Arabidopsis [48]. In plants, SKP1/CULLIN1/F-Box protein (SCF)-type E3 ubiquitin ligases are essential for the perception of auxin. The *S*-nitrosylation of TIR1 and SKP1 enhances protein–protein interactions to control the expression of auxin-responsive genes [49,50]. The *S*-nitrosylation of IAA17 at Cys-70 inhibits its interaction with TIR1, thereby negatively regulating auxin signaling [51]. These studies provide unique molecular insights into the redox-based auxin signaling in plant growth and development.

#### 3.1.3. Other Hormone Signaling Pathways

*S*-nitrosylation also participates in other hormone signaling pathways. For instance, the *S*-nitrosylation of RGA represses gibberellin signaling to balance growth and stress responses [52]. The *S*-nitrosylation of AHP1 at Cys-115 negatively regulates cytokinin signaling by inhibiting its phosphorelay activity [16]. Meanwhile, NO inhibits ABA signaling in guard cells via the *S*-nitrosylation of OST1 [53]. Therefore, a close relationship exists between NO and phytohormones during plant growth and development. Currently, there are more studies on auxin- and ABA-related proteins than other hormone-associated proteins. Future studies should focus on the *S*-nitrosylation of ethylene-, JA-, and cytokinin-associated proteins.

**Table 2:** Plant *S*-nitrosylated proteins in the regulation of growth and development.

Species	Protein	The Detecting Techniques	Functions	Result	Reference
*Arabidopsis thaliana*	SnRK2.6	BST	Inhibits enzyme activity	Promotes seed germination	[41]
*Arabidopsis thaliana*	ABI5	BST; LC-MS/MS	Promotes ABI5 degradation	Promotes seed germination	[42]
*Arabidopsis thaliana*	MYB30	BST; LC-MS/MS	Enhances MYB30 transcriptional activity	Promotes seed germination	[45]
*Arabidopsis thaliana*	ROP2	BST	Reduces the auxin transport rate	Inhibits root growth	[48]
*Arabidopsis thaliana*	TIR1	BST	Enhances TIR1-Aux/IAA interaction	Activates auxin signaling pathway	[47]
*Arabidopsis thaliana*	SKP1	BST; LC-MS/MS	Enhances SKP1 binding to CUL1-TIR1	Enhances auxin signal transduction	[49]
*Arabidopsis thaliana*	IAA17	BST; LC-MS/MS; DAN Assay	Inhibits its interaction with TIR1	Negatively regulates auxin signaling	[51]
*Arabidopsis thaliana*	RGA	BST; LC-MS/MS; DAN Assay	Inhibits its interaction with the F-box protein	Coordinates growth and stress responses	[52]
*Arabidopsis thaliana*	AHP1	BST; LC-MS/MS; DAN Assay	Inhibits AHP1 phosphorylation	Inhibits cytokinin reactions	[15]
*Arabidopsis thaliana*	OST1	BST	Inhibits OST1 activity	Negatively regulates ABA signaling	[53]

### 3.2. Immune Response

NO participates in plant immune responses, including immune-related defensive gene expression, SA-mediated immune responses, and programmed cell death (PCD) [54,55]. Crosstalk between *S*-nitrosylation and multiple nodes of immune signaling has been reported [14]. The regulatory methods of immune-related *S*-nitrosylated proteins are shown in Table 3.

#### 3.2.1. Immune-Related Gene Expression

*S*-nitrosylation can regulate the expression of immunity-related genes. For example, NO mediates the *S*-nitrosylation of two transcription factors, NPR1 and TGA1, to increase the expression of immunity-related genes [56]. The transcription factor SRG1 is a central site of NO bioactivity in plant immunity. The *S*-nitrosylation of SRG1 at Cys-87 presumably increases the transcription of immune repressors, contributing to dampening of the immune response [57]. In addition, the *S*-nitrosylation of SRG3 plays a positive role in plant immune response [58]. These studies illustrate that *S*-nitrosylation regulates immune signaling by controlling the expression of immunity-related transcription factors.

#### 3.2.2. Regulating Protein Activity

The phytohormone SA plays a critical role in plant immune signaling, helping to trigger the hypersensitivity response and activate systemic acquired resistance [59,60]. NO-induced *S*-nitrosylation supports the role of SA signaling network in immune responses. The *S*-nitrosylation of SA-binding protein 3 (AtSABP3) at Cys-280 suppresses its binding activity with the immune activator to resistant against PstDC3000 (avrB) [61]. The *S*-nitrosylation of small ubiquitin-like modifier (SUMO)-conjugating enzyme 1 (SCE1) at Cys-139 negatively regulates plant immunity by inhibiting its sumoylation [62]. NPR1, which locates in the cytoplasm as an oligomer, is a master regulator of SA-mediated defensive genes. The *S*-nitrosylation of NPR1 at Cys-156 facilitates its oligomerization to maintain protein homeostasis during plant immune response [46]. These data suggest that NO mediates the *S*-nitrosylation of SA-related proteins during defensive responses.

#### 3.2.3. Regulating Programmed Cell Death

In Arabidopsis, at the onset of the hypersensitivity response, the *S*-nitrosylation of the bacterial effector HopAI1 inhibits its phosphothreonine lyase activity to activate programmed cell death (PCD) [63]. The *S*-nitrosylation of the NADPH oxidase RBOHO regulates PCD in plant immunity by abolishing its ability to synthesize reactive oxygen intermediates [64]. The roles of *S*-nitrosylation in cellular reprogramming are emerging research direction [14].

**Table 3:** Plant *S*-nitrosylated proteins in response to immune stress.

Species	Protein	The Detecting Techniques	Functions	Results	Reference
*Arabidopsis thaliana*	NPR1/ TGA1	BST; LC-MS/MS	Enhances the DNA binding activity	Regulates plant defense response	[56]
*Arabidopsis thaliana*	SRG1	BST; LC-MS/MS	Decreases DNA binding and transcriptional inhibition activity	Attenuates the plant immune response	[57]
*Arabidopsis thaliana*	SRG3	BST	Abolishes the activity for SRG3	Positively regulates plant immunity	[58]
*Arabidopsis thaliana*	SABP3	BST; LC-MS/MS	Suppresses its binding activity	Modulates the plant defense response	[61]
*Arabidopsis thaliana*	SCE1	BST	Increases SUMO1/2 conjugate levels	Impairs the immune response	[62]
*Arabidopsis thaliana*	HopAl1	BST; DAN Assay	Inhibits phosphothreonine lyase activity	Activates cell death	[63]
*Arabidopsis thaliana*	RBOHO	BST; LC-MS/MS	Decreases enzyme activity	Disrupts cell death	[64]

### 3.3. Abiotic Stress

Plants are very sensitive to environmental changes during their life. As a result, crop yields can be dramatically affected by environmental factors [65]. Therefore, it is crucial to explore the mechanism of *S*-nitrosylation in abiotic stresses. The regulatory methods of *S*-nitrosylated proteins related to abiotic stress are summarized in Table 4.

#### 3.3.1. Improving Salt Tolerance of Plants

NO-mediated *S*-nitrosylation is an important signaling mechanism that positively regulates plant salt tolerance [1]. For instance, in *Solanum lycopersicum*, 1054 putative *S*-nitrosylated proteins involved in salt tolerance-related reactions have been identified by MS/MS [66]. Elsewhere, the *S*-nitrosylation of GAPDH increases protein activity to respond to salt stress in *Nicotiana tabacum* [67]. Further, the *S*-nitrosylation of ACOh4 promotes the synthesis of ethylene to enhance plant salt resistance [68], while the *S*-nitrosylation of PRMT5 enhances its methyltransferase activity during salt stress [34]. Recently, we reported that the *S*-nitrosylation of RAB7 regulated ion homeostasis to promote salt tolerance in Arabidopsis [69]. The *S*-nitrosylation of SIP5CR at Cys-5 increases its NAD(P)H affinity and enzymatic activity to adapt to salt stress [70]. In conclusion, *S*-nitrosylation increases plant salt tolerance by regulating different physiological functions.

#### 3.3.2. Improving Temperature Tolerance of Plants

Extreme temperature is a major abiotic stressor that limits plant growth [71]. Plants have developed various mechanisms to increase the tolerance of extreme temperature. For example, in *Ganoderma lucidum*, the NO-mediated *S*-nitrosylation of aconitase decreases the production of ROS through regulating ganoderic acid biosynthesis under heat stress [72]. In Arabidopsis, some *S*-nitrosylated proteins have been identified in response to cold stress [33]. Brassinosteroids induce protein *S*-nitrosylation to alleviate low-temperature stress in Mini Chinese Cabbage (*Brassica campestris* L.) [73]. In *Brassica juncea,* some pathogenesis-related and photosynthetic proteins have been identified to be *S*-nitrosylated under cold stress [74]. *S*-nitrosylation might increase plant tolerance to extreme temperature by reducing the level of oxidative damage.

#### 3.3.3. Improving Oxidative Tolerance of Plants

Some redox homeostasis-related proteins can be *S*-nitrosylated in plants. In Arabidopsis, the *S*-nitrosylation of hemoglobin AHb1 reduces NO emission under hypoxic stress [75]. The *S*-nitrosylation of GSNOR1 induces selective autophagy, which enhances plant tolerance to low-oxygen stress [76]. The *S*-nitrosylation of peroxidase APX1 enhances protein activity to adapt to oxidative stress [77]. These results demonstrate that *S*-nitrosylation has emerged as a connection between ROS and nitrogen species signaling. However, the crosstalk between NO and ROS requires further study.

**Table 4:** Plant *S*-nitrosylated proteins in response to abiotic stress.

Species	Protein	The Detecting Techniques	Functions	Results	Reference
*Nicotiana tabacum*	GAPDH	BST	Enhances protein kinases activity	Responses to salt stress	[67]
*Arabidopsis thaliana*	ACOh4	BST; LC-MS/MS	Promotes the synthesis of ethylene	Enhances the salt resistance	[68]
*Arabidopsis thaliana*	RAB7	BST; LC-MS/MS	Enhances protein activity	Maintains the ionic balance under salt stress	[69]
*Arabidopsis thaliana*	PRMT5	BST; LC-MS/MS; DAN Assay	Enhances methyltransferase activity	Enhances stress tolerance	[34]
*Solanum lycopersicum* L.	SIP5CR	BST	Boosts both SlP5CR activity and proline synthesis	Enhances stress tolerance	[70]
*Ganoderma lucidum*	Acon	BST; LC-MS/MS	Regulates ganoderic acid biosynthesis	Enhances heat stress	[72]
*Arabidopsis thaliana*	AHb1	BST	Reduces NO emission	Enhances hypoxic stress	[75]
*Arabidopsis thaliana*	APX1	BST; LC-MS/MS; DAN Assay	Enhances APX1 activity	Enhances the antioxidative capacity	[77]
*Arabidopsis thaliana*	GSNOR	BST; LC-MS/MS; DAN Assay;	Induces the autophagic degradation of GSNOR	Enhances hypoxic stress	[76]
*Helianthus annuus*	GAPDH	BST; LC-MS/MS	Regulates protein activity	Responses to salt stress	[78]
*Helianthus annuus*	MDHAR	BST; LC-MS/MS	Regulates protein activity	Responses to salt stress	[78]

NO plays a central role in abiotic stress [79]; it undergoes extensive crosstalk with other signaling molecules (e.g., ROS, hormonal signals, and epigenetic regulation) [80]. Current research on NO in abiotic stress mainly focuses on aspects such as antioxidant defense, signaling pathways, stomatal movement and osmotic regulatory substances [81]. However, the study of the spatial distribution of NO is still scarce. In the future, it is necessary to integrate molecular biology, nanotechnology and artificial intelligence to investigate precise NO signaling networks, providing new strategies for sustainable agriculture.

### 3.4. Role of S-Nitrosylation in Legumes

It has been reported that *S*-nitrosylated proteins exists in legumes [82]. Glutathione peroxidases (Gpxs) are highly expressed in nodules of *Lotus japonicus*. The *S*-nitrosylation of LjGpx1 and LjGpx3 inhibit enzyme activity to realize antioxidative functions [83]. In *Medicago truncatula*, the activity of plastid glutamine synthetase was inhibited by *S*-nitrosylation [84]. These results imply that *S*-nitrosylation plays a role in symbiotic nitrogen fixation. However, the mechanism of *S*-nitrosylation in symbiosis still needs more research.

## 4. Denitrosylation and Transnitrosylation

It has been reported that certain proteins have denitrosylase activities. Denitrosylation refers to the process by which a nitrosyl group (-NO) is removed from a Cys residue of an *S*-nitrosylated protein. Emerging evidence also indicates that a small group of *S*-nitrosylated proteins can transfer their NO moiety to another protein and nitrosylate the latter in a process termed transnitrosylation [85]. Several enzymes, including hemoglobin, superoxide dismutase 1, *S*-nitrosoglutathione reductase, and protein-disulfide isomerase, have been shown to possess either transnitrosylase or denitrosylase activities [86]. The discovery of denitrosylases and transnitrosylases have revealed an enzyme-based mechanism vital for the specificity of NO signaling.

Thiol-disulfide oxidoreductase thioredoxin h5 (TRXh5) can denitrosylate NPR1, which is thought to facilitate monomerization by preventing the formation of intermolecular disulfide bonds. Additionally, evidence suggests that TRXh5 is a potent protein-SNO denitrosylase with a wide set of endogenous targets [87]. ROG1 transnitrosylates GSNOR1 to promote its degradation, thereby regulating NO signaling and eventually various physiological responses [88]. Unlike other types of protein modification enzymes, identified denitrosylases and transnitrosylases remain largely uncharacterized due to a lack of consensus around conserved functional domains. Therefore, it is important to identify and characterize the adjustment mechanisms of denitrosylation and transnitrosylation.

## 5. The Crosstalk Between *S*-Nitrosylation and Other Protein PTMs

*S*-nitrosylation can regulate other protein PTMs, including phosphorylation, acetylation, ubiquitylation, and methylation, as part of its involvement in plant physiological processes. In *Arabidopsis*, the *S*-nitrosylation of PRMT5 enhances its methyltransferase activity in abiotic stress [34]. It has been reported that *S*-nitrosylation represses the phosphorylation of cytokinin signaling pathway but increases histone acetylation through the inhibition of histone deacetylases during stress responses [16,89].

Previous studies showed a crosstalk between *S*-nitrosylation and other redox-related modifications. It was reported that 639 proteins could be both persulfidated and *S*-nitrosylated (e.g., NADP-isocitrate dehydrogenase) [90]. María et al. showed that the content of H_2_S and NO increased significantly in sweet peppers during the mature period [91]. Therefore, there is an inevitable connection between H_2_S and NO in plant stress response. In addition, NO enhances the drought tolerance of *Antiaris toxicaria* seeds via protein *S*-nitrosylation and carbonylation [92]. The study has revealed that *S*-nitrosylation controls ROS and reactive nitrogen species (RNS) homeostasis in plants [93]. However, the mechanism of *S*-nitrosylation in the regulation of other protein PTMs is not clear.

## 6. Conclusions and Perspectives

**Figure 4:**
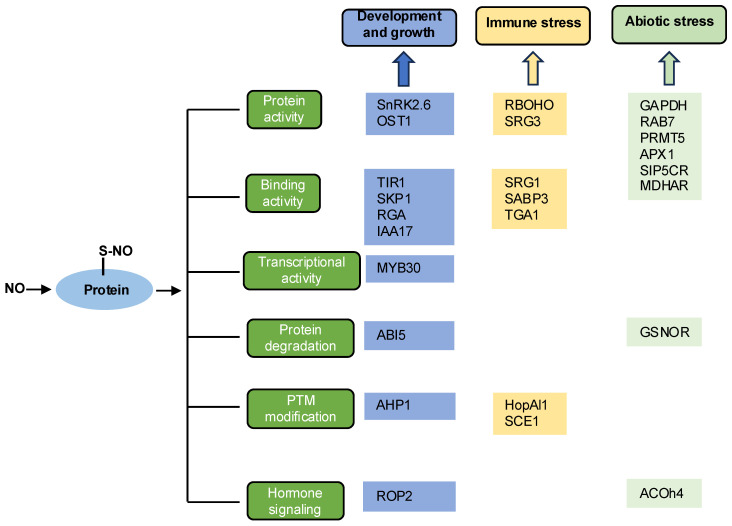
The functions of *S*-nitrosylated proteins in various physiological processes. NO-mediated protein *S*-nitrosylation affects plant physiological processes by altering protein structure and function.

In recent years, significant progress has been made in understanding the biochemical and biological functionality of protein *S*-nitrosylation. *S*-nitrosylation affects many physiological processes by controlling protein functions such as protein activity, binding activity, and transcriptional activity [8] (Figure 4). Therefore, *S*-nitrosylation plays important roles in plant biology and agricultural science.

However, the current research on *S*-nitrosylation has certain limitations and challenges. Firstly, the function of *S*-nitrosylated proteins has been widely studied in Arabidopsis, but it is rarely studied in crops [6]. A report related to *S*-nitrosylation in wheat was based on proteomic analysis and did not address the detailed role of *S*-nitrosylation in abiotic stress [94]. Since the aim of much research is to improve crop stress resistance, the application of *S*-nitrosylation to crops is crucial. Secondly, the research on *S*-nitrosylation involved in plant physiological processes is not sufficient. There are more studies on *S*-nitrosylated proteins participating in the seed germination stage than the seedling stage. Similarly, there are more studies on *S*-nitrosylated proteins related to the salt stress response than the drought stress response [95]. Therefore, further research is needed to explore extensive studies of *S*-nitrosylation related to plant growth and development. Thirdly, many proteins can be *S*-nitrosylated, but only some of the modification sites play roles during plant metabolism. Deeper research is needed to investigate the functional mechanism of *S*-nitrosylation.

Furthermore, we identified several research gaps around *S*-nitrosylation. Firstly, there is a lack of a reliable method to predict *S*-nitrosylation sites. The specific microenvironments around the *S*-nitrosylated cysteine residues (e.g., acidic residues, hydrogen bond networks) require further investigation. Secondly, it is valuable to integrate analyses of *S*-nitrosylation, transcriptomic and metabolomic data during stress response. Thirdly, there is a lack of a cross-species comparative study on *S*-nitrosylation. Such a study would uncover the conservation and diversity of *S*-nitrosylation during evolution.

The BST has been largely used because it avoids the instability of protein SNO groups. However, several limitations of this method have been reported in recent years. First, the BST is time-consuming and complicated, making it particularly inapposite for monitoring dynamic changes of *S*-nitrosylated proteins in vivo [96]. Second, the biotin-switch method is indirect and highly dependent on the complete alkylation of all free thiols. Third, *S*-nitrosylation is a low-abundance modification, so even low levels of uncapped thiols can lead to a high false-positive rate. Another source of false positives is sunlight-driven disulfide reduction, which can be eliminated by performing all procedures in complete darkness [97]. This unfortunate restraint makes sample preparation more tedious. Therefore, the development of a highly sensitive and user-friendly method should urgently be explored in the near future.

In conclusion, *S*-nitrosylation is a promising area in plants. Associated research will provide a theoretical basis for agricultural production and make an important contribution to plant biology.

## Figures and Tables

**Table 1:** The comparative table of BST and FAT-switch techniques.

	BST Techniques	FAT-Switch Techniques
Sensitivity and efficiency	Low	High
Specificity	High	High
Cost	Middle	Middle
Limitation of method	Time-consuming and tedious; Unsuitable for monitoring dynamic changes in *S*-nitrosylation	Lack of commercial antibodies; Unsuitable for monitoring dynamic changes in *S*-nitrosylation

## References

[1] Shang J.X., Li X., Li C., Zhao L. (2022). The Role of nitric oxide in plant responses to salt stress. Int. J. Mol. Sci..

[2] Khator K., Parihar S., Jasik J., Shekhawat G.S. (2024). Nitric oxide in plants: An insight on redox activity and responses toward abiotic stress signaling. Plant Signal. Behav..

[3] Khan M.N., Siddiqui M.H., Mohammad F., Naeem M. (2012). Interactive role of nitric oxide and calcium chloride in enhancing tolerance to salt stress. Nitric Oxide.

[4] Tian X., He M., Wang Z., Zhang J., Song Y., He Z., Dong Y. (2015). Application of nitric oxide and calcium nitrate enhances tolerance of wheat seedlings to salt stress. Plant Growth Regul..

[5] Santos M.P., Zandonadi D.B., de Sa A.F.L., Costa E.P., de Oliveira C.J.L., Perez L.E.P., Façanha A.R., Bressan-Smith R. (2020). Abscisic acid-nitric oxide and auxin interaction modulates salt stress response in tomato roots. Theor. Exp. Plant Physiol..

[6] Feng J., Chen L., Zuo J. (2019). Protein *S*-nitrosylation in plants: Current progresses and challenges. J. Integr. Plant Biol..

[7] Hess D.T., Matsumoto A., Kim S.O., Marshall H.E., Stamler J.S. (2005). Protein *S*-nitrosylation: Purview and parameters. Nat. Rev. Mol. Cell Biol..

[8] Hess D.T., Stamler J.S. (2012). Regulation by *S*-nitrosylation of protein post-translational modification. J. Biol. Chem..

[9] López-Sánchez L.M., López-Pedrera C., Rodríguez-Ariza A. (2012). Proteomics insights into deregulated protein *S*-nitrosylation and disease. Expert. Rev. Proteom..

[10] Zavadskiy S., Sologova S., Moldogazieva N. (2022). Oxidative distress in aging and age-related diseases: Spatiotemporal dysregulation of protein oxidation and degradation. Biochimie.

[11] Marino S.M., Gladyshev V.N. (2010). Structural analysis of cysteine *S*-nitrosylation: A modified acid-based motif and the emerging role of trans-nitrosylation. Nat. Commun..

[12] Zhang C., Xu Y., Wang G., Fang C., Bao H., Zhang Y., Lu H. (2020). FluoroTRAQ: Quantitative analysis of protein *S*-nitrosylation through Fuorous solid-phase extraction combining with iTRAQ by mass spectrometry. Anal. Chem..

[13] Qin G., Qu M., Jia B., Wang W., Luo Z., Song C.P., Tao W.A., Wang P. (2023). FAT-switch-based quantitative *S*-nitrosoproteomics reveals a key role of GSNOR1 in regulating ER functions. Nat. Commun..

[14] Borrowman S., Kapuganti J.G., Loake G.J. (2023). Expanding roles for nitrosylation in the regulation of plant immunity. Free Radic. Biol. Med..

[15] Jaffrey S.R., Snyder S.H. (2001). The biotin switch method for the detection of *S*-nitrosylated proteins. Sci. STKE.

[16] Feng J., Wang C., Chen Q., Chen H., Ren B., Li X., Zuo J. (2013). *S*-nitrosylation of phosphotransfer proteins represses cytokinin signaling. Nat. Commun..

[17] Zhang Y., Keszler A., Broniowska K.A., Hogg N. (2005). Characterization and application of the biotin-switch assay for the identification of *S*-nitrosated proteins. Free Radic. Biol. Med..

[18] Wu C., Parrott A.M., Liu T., Jain M.R., Yang Y., Sadoshima J., Li H. (2011). Distinction of thioredoxin transnitrosylation and denitrosylation target proteins by the ICAT quantitative approach. J. Proteom.

[19] Qu Z., Meng F., Bomgarden R.D., Viner R.I., Li J., Rogers J.C., Cheng J., Greenlief C.M., Cui J., Lubahn D.B. (2014). Proteomic quantification and site-mapping of *S*-nitrosylated proteins using isobaric iodoTMT reagents. J. Pro-Teome Res..

[20] Gong B., Shi Q. (2019). Identifying *S*-nitrosylated proteins and unraveling *S*-nitrosoglutathione reductase-modulated sodic alkaline stress tolerance in *Solanum lycopersicum* L.. Plant Physiol Biochem..

[21] Ibáñez-Vea M., Huang H., Martínez X., Pérez E., Gato M., Zuazo M., Arasanz H., Fernández-Irigoyen J., Santamaría E., Fernandez-Hinojal G. (2018). Characterization of macrophage endogenous *S*-nitrosoproteome using a cysteine-specific phosphonate adap-table tag in combination with TiO_2_ Chromatography. J. Proteome Res..

[22] Mnatsakanyan R., Markoutsa S., Walbrunn K., Roos A., Verhelst S.H.L., Zahedi R.P. (2019). Proteome-wide detection of *S*-nitrosylation targets and motifs using bioorthogonal cleavable-linker-based enrichment and switch technique. Nat. Commun..

[23] Forrester M.T., Thompson J.W., Foster M.W., Nogueira L., Moseley M.A., Stamler J.S. (2009). Proteomic analysis of *S*-nitrosylation and denitrosylation by resin-assisted capture. Nat. Biotechnol.

[24] Kim J.K., Lee J.R., Kang J.W., Lee S.J., Shin G.C., Yeo W.S., Kim K.H., Park H.S., Kim K.P. (2011). Selective enrichment and mass spectrometric identification of nitrated peptides using fluorinated carbon tags. Anal. Chem..

[25] Zhao M., Deng C. (2016). Designed synthesis of fluorous-functionalized magnetic. mesoporous microspheres for specific enrichment of phosphopeptides with fluorous derivatization. Proteomics.

[26] Foster M.W., Forrester M.T., Stamler J.S. (2009). A protein microarray-based analysis of *S*-nitrosylation. Proc. Natl. Acad. Sci. USA.

[27] Cho D.H., Nakamura T., Fang J., Cieplak P., Godzik A., Gu Z., Lipton S.A. (2009). *S*-nitrosylation of Drp1 mediates beta-amyloid- related mitochondrial fission and neuronal injury. Science.

[28] Hu J., Huang X., Chen L., Sun X., Lu C., Zhang L., Wang Y., Zuo J. (2015). Site-specific nitrosoproteomic identification of endogenously *S*-nitrosylated proteins in Arabidopsis. Plant Physiol..

[29] Silva A.M., Vitorino R., Domingues M.R., Spickett C.M., Domingues P. (2013). Post-translational modifications and mass spectrometry detection. Free Radic. Biol. Med..

[30] Chicooree N., Unwin R.D., Griffiths J.R. (2014). The application of targeted mass spectrometry-based strategies to the detection and localization of post- translational modifications. Mass Spectrom. Rev..

[31] Astier J., Besson-Bard A., Lamotte O., Bertoldo J., Bourque S., Terenzi H., Wendehenne D. (2012). Nitric oxide inhibits the ATPase activity of the chaperone-like AAA^+^ ATPase CDC48, a target for *S*-nitrosylation in cryptogein signalling in tobacco cells. Biochem. J..

[32] Chaki M., Kovacs I., Spannagl M., Lindermayr C. (2014). Computational prediction of candidate proteins for *S*-nitrosylation in Arabidopsis thaliana. PLoS ONE.

[33] Puyaubert J., Fares A., Rézé N., Peltier J.B., Baudouin E. (2014). Identification of endogenously *S*-nitrosylated proteins in Arabidopsis plantlets: Effect of cold stress on cysteine nitrosylation level. Plant Sci..

[34] Hu J., Yang H., Mu J., Lu T., Peng J., Deng X., Kong Z., Bao S., Cao X., Zuo J. (2017). Nitric oxide regulates protein methylation during stress responses in Plants. Mol. Cell.

[35] Sánchez-Vicente I., Fernández-Espinosa M.G., Lorenzo O. (2019). Nitric oxide molecular targets: Reprogramming plant development upon stress. J. Exp. Bot..

[36] Sanchez-Corrionero A., Sánchez-Vicente I., Arteaga N., Manrique-Gil I., Gómez-Jiménez S., Torres-Quezada I., Albertos P., Lorenzo O. (2023). Fine-tuned nitric oxide and hormone interface in plant root development and regeneration. J. Exp. Bot..

[37] Tripathi D.K., Bhat J.A., Ahmad P., Allakhverdiev S.I. (2023). Polyamines and nitric oxide crosstalk in plant development and abiotic stress tolerance. Funct. Plant Biol. FPB.

[38] Ali F., Qanmber G., Li F., Wang Z. (2021). Updated role of ABA in seed maturation, dormancy, and germination. J. Adv. Res..

[39] Beligni M.V., Lamattina L. (2000). Nitric oxide stimulates seed germination and de-etiolation, and inhibits hypocotyl elongation, three light-inducible responses in plants. Planta.

[40] Bethke P.C., Gubler F., Jacobsen J.V., Jones R.L. (2004). Dormancy of Arabidopsis seeds and barley grains can be broken by nitric oxide. Planta.

[41] Wang P., Zhu J.K., Lang Z. (2015). Nitric oxide suppresses the inhibitory effect of abscisic acid on seed germination by *S*-nitrosylation of SnRK2 proteins. Plant Signal. Behav..

[42] Albertos P., Romero-Puertas M.C., Tatematsu K., Mateos I., Sánchez-Vicente I., Nambara E., Lorenzo O. (2015). *S*-nitrosylation triggers ABI5 degradation to promote seed germination and seedling growth. Nat. Commun..

[43] Zhao H., Zhang Y., Zheng Y. (2022). Integration of ABA, GA, and light signaling in seed germination through the regulation of ABI5. Front. Plant Sci..

[44] Nie K., Zhao H., Wang X., Niu Y., Zhou H., Zheng Y. (2022). The MIEL1-ABI5/MYB30 regulatory module fine tunes abscisic acid signaling during seed germination. J. Integr. Plant Biol..

[45] Zhao H., Ma L., Shen J., Zhou H., Zhen Y. (2024). *S*-nitrosylation of the transcription factor MYB30 facilitates nitric oxide-promoted seed germination in Arabidopsis. Plant Cell.

[46] Tada Y., Spoel S.H., Pajerowska-Mukhtar K., Mou Z., Song J., Wang C., Zuo J., Dong X. (2008). Plant immunity requires conformational charges of NPR1 via *S*-nitrosylation and thioredoxins. Science.

[47] Terrile M.C., París R., Calderón-Villalobos L.I., Iglesias M.J., Lamattina L., Estelle M., Casalongué C.A. (2012). Nitric oxide influences auxin signaling through *S*-nitrosylation of the Arabidopsis TRANSPORT INHIBITOR RESPONSE 1 auxin receptor. Plant J..

[48] Kenesi E., Kolbert Z., Kaszler N., Klement E., Menesi D., Molnar A., Valkai I., Feigl G., Rigó G., Cséplő A. (2023). The ROP2 GTPase participates in nitric oxide (NO)-induced root shortening in Arabidopsis. Plants.

[49] Iglesias M.J., Terrile M.C., Correa-Aragunde N., Colman S.L., Izquierdo-Álvarez A., Fiol D.F., París R., Sánchez-López N., Marina A., Villalobos L.I.A.C. (2018). Regulation of SCFTIR1/AFBs E3 ligase assembly by *S*-nitrosylation of Arabidopsis SKP1-like1 impacts on auxin signaling. Redox Biol..

[50] Terrile M.C., Tebez N.M., Colman S.L., Mateos J.L., Morato-Lopez E., Sanchez-Lopez N., Izquierdo-Álvarez A., Marina A., Villalobos L.I.A.C., Estelle M. (2021). *S*-nitrosation of E3 ubiquitin ligase complex components regulates hormonal signalings in Arabidopsis. Front. Plant Sci..

[51] Jing H., Yang X., Emenecker R.J., Feng J., Zhang J., Figueiredo M.R.A., Chaisupa P., Wright R.C., Holehouse A.S., Strader L.C. (2023). Nitric oxide-mediated *S*-nitrosylation of IAA17 protein in intrinsically disordered region represses auxin signaling. J. Genet. Genom..

[52] Chen L., Sun S., Song C.P., Zhou J.M., Li J., Zuo J. (2022). Nitric oxide negatively regulates gibberellin signaling to coordinate growth and salt tolerance in Arabidopsis. J. Genet. Genom..

[53] Wang P., Du Y., Hou Y.J., Zhao Y., Hsu C.C., Yuan F., Zhu X., Tao W.A., Song C.P., Zhu J.K. (2015). Nitric oxide negatively regulates abscisic acid signaling in guard cells by *S*-nitrosylation of OST1. Proc. Natl. Acad. Sci. USA.

[54] Yun B.W., Skelly M.J., Yin M., Yu M., Mun B.G., Lee S.U., Hussain A., Spoel S.H., Loake G.J. (2016). Nitric oxide and *S*-nitrosoglutathione function additively during plant immunity. New Phytol..

[55] Imran Q.M., Hussain A., Lee S.U., Mun B.G., Falak N., Loake G.J., Yun B.W. (2018). Transcriptome profile of NO-induced Arabidopsis transcription factor genes suggests their putative regulatory role in multiple biological processes. Sci. Rep..

[56] Lindermayr C., Sell S., Müller B., Leister D., Durner J. (2010). Redox regulation of the NPR1-TGA1 system of *Arabidopsis thaliana* by nitric oxide. Plant Cell.

[57] Cui B., Pan Q., Clarke D., Villarreal M.O., Umbreen S., Yuan B., Shan W., Jiang J., Loake G.J. (2018). *S*-nitrosylation of the zinc finger protein SRG1 regulates plant immunity. Nat. Commun..

[58] Cui B., Xu S., Li Y., Umbreen S., Frederickson D., Yuan B., Jiang J., Liu F., Pan Q., Loake G.J. (2021). The Arabidopsis zinc finger proteins SRG2 and SRG3 are positive regulators of plant immunity and are differentially regulated by nitric oxide. New Phytol..

[59] Loake G., Grant M. (2007). Salicylic acid in plant defence—The players and protagonists. Curr. Opin. Plant Biol..

[60] Fu Z.Q., Dong X. (2013). Systemic acquired resistance: Turning local infection into global defense. Plant Biol..

[61] Wang Y.Q., Feechan A., Yun B.W., Shafiei R., Hofmann A., Taylor P., Xue P., Yang F.Q., Xie Z.S., Pallas J.A. (2009). *S*-nitrosylation of AtSABP3 antagonizes the expression of plant immunity. J. Biol. Chem..

[62] Skelly M.J., Malik S.I., Le Bihan T., Bo Y., Jiang J., Spoel S.H., Loake G.J. (2019). A role for *S*-nitrosylation of the SUMO-conjugating enzyme SCE1 in plant immunity. Proc. Natl. Acad. Sci. USA.

[63] Ling T., Bellin D., Vandelle E., Imanifard Z., Delledonne M. (2017). Host-mediated *S*-nitrosylation disarms the bacterial effec- tor HopAI1 to reestablish immunity. Plant Cell.

[64] Yun B.W., Feechan A., Yin M., Saidi N.B., Le Bihan T., Yu M., Moore J.W., Kang J.G., Kwon E., Spoel S.H. (2011). *S*-nitrosylation of NADPH oxidase regulates cell death in plant immunity. Nature.

[65] Saddhe A.A., Malvankar M.R., Karle S.B., Kumar K. (2018). Reactive nitrogen species: Paradigms of cellular signaling and regulation of salt stress in plants. Environ. Exp. Bot..

[66] Wei L., Zhang J., Wei S., Wang C., Deng Y., Hu D., Liu H., Gong W., Pan Y., Liao W. (2022). Nitric oxide alleviates salt stress through protein *S*-nitrosylation and transcriptional regulation in tomato seedlings. Planta.

[67] Wawer I., Bucholc M., Astier J., Anielska-Mazur A., Dahan J., Kulik A., Wysłouch-Cieszynska A., Zaręba-Kozioł M., Krzywinska E., Dadlez M. (2010). Regulation of *Nicotiana tabacum* osmotic stress-activated protein kinase and its cellular partner GAPDH by nitric oxide in response to salinity. Biochem. J..

[68] Liu M., Wei J.W., Liu W., Gong B. (2023). *S*-nitrosylation of ACO homolog 4 improves ethylene synthesis and salt tolerance in tomato. New Phytol..

[69] Lin W., Wang Y., Li X., Huang X., Wang Y., Shang J.X., Zhao L. (2023). *S*-nitrosylation of RABG3E positively regulates vesicle trafficking to promote salt tolerance. Plant Cell Environ..

[70] Liu W., Wei J.W., Shan Q., Liu M., Xu J., Gong B. (2024). Genetic engineering of drought- and salt-tolerant tomato via Δ1-pyrroline-5-carboxylate reductase *S*-nitrosylation. J. Integr. Plant Biol..

[71] Li B., Gao K., Ren H., Tang W. (2018). Molecular mechanisms governing plant responses to high temperatures. J. Integr. Plant Biol..

[72] Liu R., Zhu T., Yang T., Yang Z., Ren A., Shi L., Zhu J., Yu H., Zhao M. (2021). Nitric oxide regulates ganoderic acid biosynthesis by the *S*-nitrosylation of aconitase under heat stress in *Ganoderma lucidum*. Environ. Microbiol..

[73] Gao X., Ma J., Tie J., Li Y., Hu L., Yu J. (2022). BR-Mediated protein *S*-nitrosylation alleviated low-temperature stress in Mini Chinese cabbage (*Brassica rapa* ssp. *pekinensis*). Int. J. Mol. Sci..

[74] Abat J.K., Deswal R. (2009). Differential modulation of *S*-nitrosoproteome of *Brassica juncea* by low temperature: Change in *S*-nitrosylation of Rubisco is responsible for the inactivation of its carboxylase activity. Proteomics.

[75] Perazzolli M., Dominici P., Romero-Puertas M.C., Zago E., Zeier A., Sonoda M., Lamb C., Delledonne M. (2004). Arabidopsis nonsymbiotic hemoglobin AHb1 modulates nitric oxide bioactivity. Plant Cell.

[76] Zhan N., Wang C., Chen L., Yang H., Feng J., Gong X., Ren B., Wu B., Mu J., Li Y. (2018). *S*-nitrosylation targets GSNO reductase for selective autophagy during hypoxia responses in plants. Mol. Cell.

[77] Yang H., Mu J., Chen L., Feng J., Hu J., Li L., Zhou J.M., Zuo J. (2015). *S*-nitrosylation positively regulates ascorbate peroxidase activity during plant stress responses. Plant Physiol..

[78] Jain P., von Toerne C., Lindermayr C., Bhatla S.C. (2018). *S*-nitrosylation/denitrosylation as a regulatory mechanism of salt stress sensing in sunflower seedlings. Plant Physiol..

[79] Allagulova C.R., Lubyanova A.R., Avalbaev A.M. (2023). Multiple ways of nitric oxide production in plants and its functional activity under abiotic stress conditions. Int. J. Mol. Sci..

[80] Zhou X., Joshi S., Khare T., Patil S., Shang J., Kumar V. (2021). Nitric oxide, crosstalk with stress regulators and plant abiotic stress tolerance. Plant Cell Rep..

[81] Hasanuzzaman M., Oku H., Nahar K., Borhannuddin Bhuyan M.H.M., Mahmud J., Baluska F., Fujita M. (2018). Nitric oxide-induced salt stress tolerance in plants: ROS metabolism, signaling, and molecular interactions. Plant Biotechnol. Rep..

[82] Matamoros M.A., Becana M. (2021). Molecular responses of legumes to abiotic stress: Post-translational modifications of proteins and redox signaling. J. Exp. Bot..

[83] Matamoros M.A., Saiz A., Peñuelas M., Bustos-Sanmamed P., Mulet J.M., Barja M.V., Rouhier N., Moore M., James E.K., Dietz K.J. (2015). Function of glutathione peroxidases in legume root nodules. J. Exp. Bot..

[84] Melo P.M., Silva L.S., Ribeiro I., Seabra A.R., Carvalho H.G. (2011). Glutamine synthetase is a molecular target of nitric oxide in root nodules of Medicago truncatula and is regulated by tyrosine nitration. Plant Physiol..

[85] Benhar M., Forrester M., Stamler J. (2009). Protein denitrosylation: Enzymatic mechanisms and cellular functions. Nat. Rev. Mol. Cell Biol..

[86] Wu C., Liu T., Chen W., Oka S., Fu C., Jain M.R., Parrott A.M., Baykal A.T., Sadoshima J., Li H. (2010). Redox regulatory mechanism of transnitrosylation by thioredoxin. Mol. Cell Proteom..

[87] Kneeshaw S., Gelineau S., Tada Y., Loake G.J., Spoel S.H. (2014). Selective protein denitrosylation activity of thioredoxin-h5 modulates plant immunity. Mol. Cell.

[88] Chen L., Wu R., Feng J., Feng T., Wang C., Hu J., Zhan N., Li Y., Ma X., Ren B. (2020). Transnitrosylation mediated by the non-canonical catalase ROG1 regulates nitric oxide signaling in plants. Dev. Cell.

[89] Mengel A., Ageeva A., Georgii E., Bernhardt J., Wu K., Durner J., Lindermayr C. (2017). Nitric oxide modulates histone acetylation at stress genes by inhibition of histone deacetylases. Plant Physiol..

[90] Wang P., Fang H., Gao R., Liao W. (2021). Protein persulfidation in plants: Function and mechanism. Antioxidants.

[91] Munoz-Vargas M.A., Gonzalez-Gordo S., Canas A., Lopez-Jaramillo J., Palma J.M., Corpas F.J. (2018). Endogenous hydrogen sulfide (H_2_S) is up-regulated during sweet pepper (*Capsicum annuum* L.) fruit ripening. In vitro analysis shows that NADP-dependent isocitrate dehydrogenase (ICDH) activity is inhibited by H_2_S and NO. Nitric Oxide.

[92] Bai X., Yang L., Tian M., Chen J., Shi J., Yang Y., Hu X. (2011). Nitric oxide enhances desiccation tolerance of recalcitrant *Antiaris toxicaria* seeds via protein *S*-nitrosylation and carbonylation. PLoS ONE.

[93] Wang Y., Chu C. (2020). *S*-nitrosylation control of ROS and RNS homeostasis in plants: The switching function of catalase. Mol. Plant.

[94] Gietler M., Nykiel M., Orzechowski S., Fettke J., Zagdańska B. (2016). Proteomic analysis of *S*-nitrosylated and *S*-glutathionylated proteins in wheat seedlings with different dehydration tolerances. Plant Physiol. Biochem..

[95] Begara-Morales J.C., Chaki M., Valderrama R., Mata-Pérez C., Padilla M.N., Barroso J.B. (2019). The function of *S*-nitrosothiols during abiotic stress in plants. J. Exp. Bot..

[96] Huang B., Chen C. (2010). Detection of protein *S*-nitrosation using irreversible biotinylation procedures (IBP). Free Radic. Biol. Med..

[97] Forrester M.T., Foster M.W., Stamler J.S. (2007). Assessment and application of the biotin switch technique for examining protein *S*-nitrosylation under conditions of pharmacologically induced oxidative stress. J. Biol. Chem..

